# Prevalence and Correlates of Dietary Supplement Use Among Italian University Students: A Cross-Sectional Study

**DOI:** 10.3390/nu18172760

**Published:** 2026-08-24

**Authors:** Francesco Leonforte, Vito Nicosia, Michelangelo Mercogliano, Andrea Cavallaro, Giustino Morlino, Martina Chimienti, Roberta Granata, Antonio Mistretta, Massimiliano Veroux

**Affiliations:** 1 Department of Integrated Hygiene, Organizational, and Service Activities (Structural Department), Health Management, University Hospital Polyclinic “G. Rodolico-San Marco”, 95123 Catania, Italy; 2 Department of Medical, Surgical Sciences and Advanced Technologies “G.F. Ingrassia”, University of Catania, 95123 Catania, Italy; vitonicosia6@gmail.com (V.N.); robertagranat@live.it (R.G.); anmist@unict.it (A.M.); veroux@unict.it (M.V.); 3 Department of Public Health, University “Federico II” of Naples, 80131 Naples, Italy; 4 General Surgery III, Department of General Surgery and Medical-Surgical Specialties, University of Catania, AOU Policlinico “G. Rodolico-San Marco”, 95123 Catania, Italy; andrea.cavallaro@unict.it; 5 Department of Biomedicine and Prevention, University of Rome Tor Vergata, 00133 Rome, Italy; gmiustus@gmail.com (G.M.); martina.chimienti@gmail.com (M.C.); 6 Scientific Communication Service, National Institute of Public Health (Istituto Superiore di Sanità), 00161 Rome, Italy

**Keywords:** dietary supplements, university students, health literacy, public health, adverse effects, nutritional education

## Abstract

**Background/Objectives**: Dietary supplement (DS) use is a growing trend among university students, often influenced by non-institutional information. This study investigated the lifetime prevalence, behavioural correlates, and self-reported adverse effects of DS use according to EU regulatory definitions, evaluating how consumption frequency and product diversity relate to safety outcomes and student receptivity toward educational initiatives. **Methods**: A cross-sectional survey was conducted among 2001 students at the University of Catania. Multivariable logistic regression models were utilized to identify factors associated with lifetime DS use and favourability toward informative meetings. **Results**: The lifetime prevalence of DS use was 55.7%. The strongest correlate of use was self-rated knowledge, with comparable odds among moderately (OR = 4.02; 95% CI: 3.17–5.10) and well/completely informed participants (OR = 4.70; 95% CI: 3.44–6.42). Increased odds of consumption were also associated with information from health professionals (OR = 2.19; 95% CI: 1.59–3.02), friends (OR = 1.91; 95% CI: 1.37–2.65), and enrollment in scientific fields (OR = 1.70; 95% CI: 1.23–2.35). Overall, 34.6% of users reported at least one symptom temporally associated with supplement intake. Despite these reports, 74.3% of the sample welcomed institutional meetings, with favourability among users significantly associated with daily supplement intake (OR = 1.97; 95% CI: 1.23–3.14) and smoking status (OR = 1.77; 95% CI: 1.24–2.54). **Conclusions**: The results highlight a gap between high self-rated knowledge and the frequency of self-reported side effects. The strong student interest in formal guidance underscores the need for universities to integrate evidence-based nutritional and supplement-safety education into their curricula to ensure safe supplementation practices.

## 1. Introduction

Dietary supplements (DSs) are defined as concentrated sources of nutrients, such as vitamins and minerals, or of other substances having a nutritional or physiological effect, in particular, but not exclusively, amino acids, essential fatty acids, fibres and extracts of vegetable origin [1,2]. These products are marketed in dosage forms (such as capsules, tablets or sachets) and are designed to promote the regular functioning of the organism, without however replacing a balanced diet or possessing the therapeutic properties characteristic of pharmaceuticals [3,4]. According to the Directive 2002/46/EC of the European Union (EU) [2], this study defines DSs strictly as food products intended to complement the normal diet. This operational definition differs from the one adopted in the United States under the Dietary Supplement Health and Education Act (DSHEA) [5], which also encompasses botanical and herbal extracts; in the EU, pharmacologically active botanical preparations are instead regulated as medicinal products [6].

Within this regulatory framework, the use of DSs has become a growing global phenomenon, driven by a widespread perception of safety and the pursuit of psychophysical well-being, especially among younger generations [7,8]. Population-level data illustrate the scale of the phenomenon: surveys among university students report prevalences ranging from approximately one-third to more than two-thirds across countries [9,10,11,12], while the Italian multicentre DiSCo study reported use in the previous six months by 71.5% of 2165 undergraduates [13]. In the university context, this trend takes on particular relevance, as students represent a target vulnerable to the influence of digital media and the pressures related to physical and academic performance [14,15].

Despite this widespread use, several gaps remain regarding safe consumption and health literacy. Information on supplements is most often obtained from informal channels, such as the internet, social media and fitness trainers, rather than from healthcare professionals [16,17], and symptoms attributed to supplementation are reported by a non-negligible minority of consumers [10,18]. In this scenario, unsupervised nutritional integration raises critical questions about the real awareness of users regarding potential health risks and the scientific basis justifying their intake [19]. International scientific literature highlights a growing concern regarding the discrepancy between the wide distribution of such products and the quality of information on which students base their purchasing decisions [16,20]. Consumption is often mediated by non-institutional information channels, such as social media or word-of-mouth in sports environments, which can promote irrational or excessive use to the detriment of professional advice [17,21]. The powerful role of digital media exposure in shaping health attitudes and potentially fostering misinformation is a systemic issue, widely documented in other public health contexts [17]. This dynamic is further complicated by the fallacious perception of supplements as intrinsically safe substances because they are “natural”, leading to an underestimation of possible adverse effects or pharmacological interactions [18,22].

Consequently, despite the relevance of the phenomenon, data regarding the correlation between self-reported knowledge, the information sources used, and actual consumption patterns remain partial, especially within the Italian academic context [13]. It is therefore essential to analyze not only the prevalence of use but also the socio-demographic and academic factors associated with students’ attitudes toward dietary integration [23]. Furthermore, assessing the receptivity of the student population toward institutional training interventions is a key step for structuring future public health strategies within universities [24].

Based on these premises, this cross-sectional study aims to investigate the factors associated with the lifetime use of DSs. The analysis focuses on the stratification between users and non-users, exploring differences in terms of self-rated knowledge, privileged sources of information, and beliefs related to fitness. Finally, students’ favourability toward university-based informative initiatives is examined.

## 2. Materials and Methods

This cross-sectional study was conducted among students at the University of Catania between September and October 2025. The local Ethics Committee of Catania 1 approved the study protocol (Protocol no. 55462, no. 56/2025-PAR). Participation was voluntary and electronic informed consent was obtained from all participants prior to data collection. Data were gathered using an anonymous, self-administered web-based questionnaire, provided in the Supplementary Material File S1, disseminated as an open link through a QR code and class student groups; participants were therefore recruited by convenience sampling. Inclusion criteria, verified through mandatory screening items at the beginning of the questionnaire, were informed consent; age 18–45 years; and current enrolment at the University of Catania. Exclusion criteria were pregnancy; a previously diagnosed eating disorder; and chronic conditions affecting diet, physical activity or supplement intake.

### 2.1. Variables

The main outcome was the lifetime use of DSs, coded as a binary variable (yes/no) and assessed through a single gateway item; participants reporting use were then asked follow-up items referring to the preceding 12 months. As this study was conducted within the European Union, subject to EU food supplement regulation, supplements were categorized into the following self-reported types: protein powders, creatine, amino acids/BCAA, pre-/post-workout products, mass gainers, vitamin/mineral supplements, and thermogenics. As these data were collected via a self-administered questionnaire, this classification relies on self-declaration.

Sociodemographic covariates included age group, sex, smoking status, degree course level, and field of study. Additionally, the following variables were assessed: source of information on supplement-related risks, self-rated knowledge regarding DS use, belief that supplements are necessary for fitness, and favourability towards university-based informative meetings on DS use and risks. The last one was also the outcome for additional analysis. For participants reporting lifetime DS use, additional use-related variables referring to the preceding 12 months were collected: number of supplement types used (1–2 vs. 3–7), any self-reported symptoms, frequency of use and the source of the recommendation. Symptoms were recorded through a multiple-response item listing gastrointestinal complaints, headache, insomnia, palpitations or tachycardia, mood alterations, and renal or hepatic problems, together with a “no symptoms” option. These symptoms were self-reported.

### 2.2. Statistical Analysis

Descriptive statistics were used to summarize participants’ characteristics, DS use, and opinion-related variables. Categorical variables were reported as frequencies and percentages. Comparisons were performed using Pearson’s chi-squared test, as appropriate.

To investigate factors associated with lifetime DS use, a multivariable logistic regression model was fitted. The model controlled for self-rated knowledge, information sources, fitness beliefs, and favourability towards informative meetings. Adjustments were made for age group, sex, smoking status, degree course level, and field of study.

Further subgroup analyses were conducted separately for DS users and non-users. In these models, the outcome was the favourability towards university-based informative meetings. Models included self-rated knowledge, information source, fitness beliefs, and all sociodemographic covariates. Only among users, the model was additionally adjusted for the number of supplement types, self-reported side effects, and frequency of use.

In the multivariable models, the original categories of self-rated knowledge on supplement use were combined into three analytically meaningful levels (poorly/not informed, moderately informed, and well/completely informed) to avoid sparse-data issues, increase the robustness of the regression estimates, and enhance interpretability.

Results are reported as odds ratios (ORs) with 95% confidence intervals (95% CIs). A *p*-value < 0.05 was considered statistically significant. All analyses were performed using Stata/MP version 18 (StataCorp LLC, College Station, TX, USA).

## 3. Results

A total of 2001 students (respondents) were included in the analysis. Respondents’ sociodemographic, academic, and opinion-related characteristics are reported in Table 1. Of these respondents, 1114 (55.7%) reported lifetime use of DSs, while 887 (44.3%) were non-users. Participants were predominantly aged 18–23 years (52.9%). The majority were enrolled in a Bachelor’s degree programme (53.5%). Scientific/technological (35.7%) and health-related disciplines (37.1%) were the most frequent fields of study. The internet and social media were the primary sources of information (34%). A large proportion of the sample (74.3%) expressed favourability towards university-based informative meetings (Table 1).

No significant differences in DS use were observed across age groups (*p* = 0.291) or degree course levels. Conversely, significant differences between users and non-users were found regarding sex, smoking status, field of study, and variables related to knowledge and opinions about DSs (*p* < 0.001). DS use was more frequent among males, smokers, and students enrolled in scientific or technological fields. Furthermore, among participants, a higher proportion of users were observed who received information on DS risks from the internet or social media, self-rated their knowledge as moderate, believed supplements are necessary for fitness, and expressed favourability towards university-based informative meetings (Table 1).

Specific characteristics of DS users are presented in Table 2. The use of one or two different supplements in the last year was reported by the majority of users (77.7%). Daily use was reported by 53.4% of them. Side effects were reported by 34.6% of supplement users; personal trainers were the most frequently reported source of recommendation for supplement use (31.7%), and only a small proportion of the sample (8.5%) received the recommendation from a physician.

A multivariable logistic regression model was performed to identify factors associated with lifetime DS use, adjusting for selected sociodemographic variables. Lifetime use was positively associated with moderate self-rated knowledge (OR = 4.0; 95% CI: 3.2–5.1) and for well or completely informed participants (OR = 4.7; 95% CI: 3.4–6.4). Supplement use was also associated with favourability towards university-based informative meetings (OR = 1.9; 95% CI: 1.5–2.4) and with the belief that supplements are necessary for fitness (OR = 1.6; 95% CI: 1.3–2.0).

**Figure 1:**
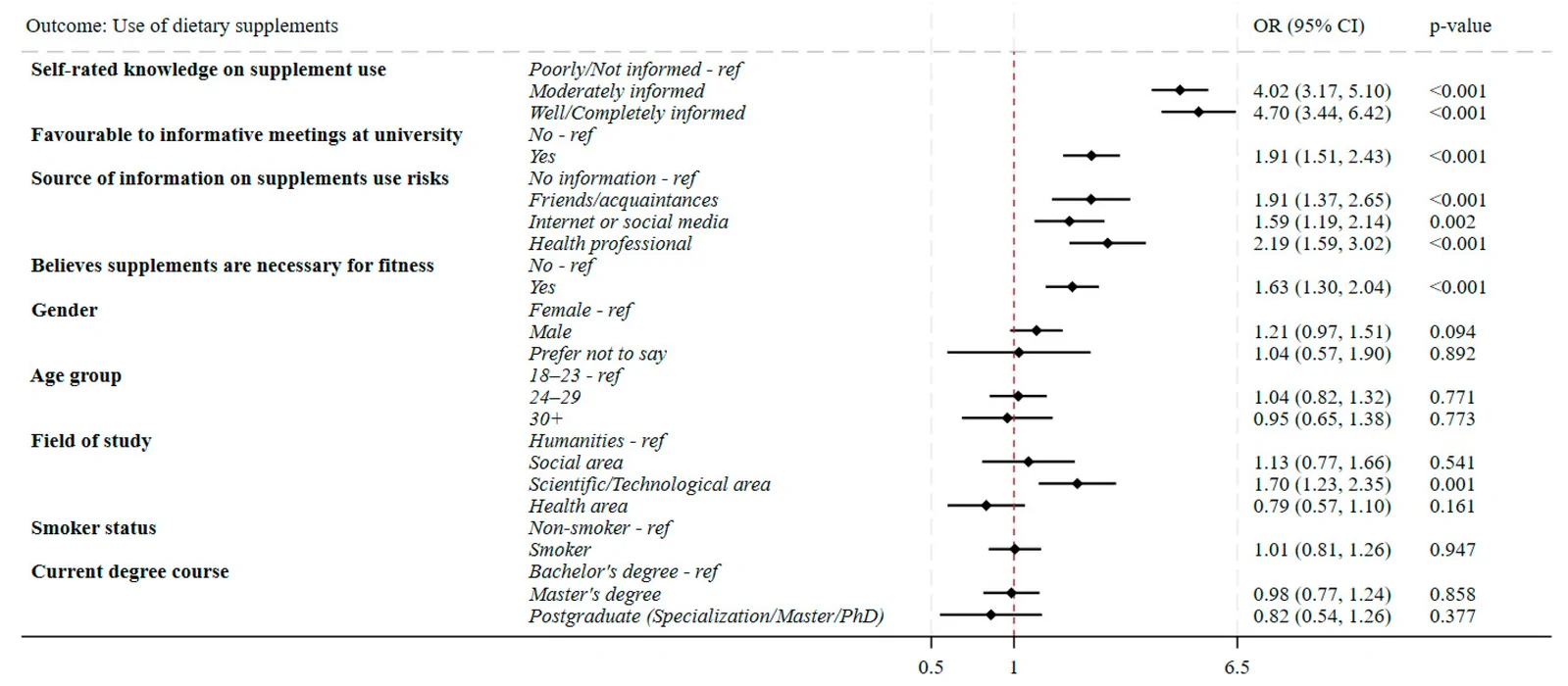
Multivariable logistic regression analysis of factors associated with lifetime dietary supplement use (full sample of respondents). Data are presented as adjusted odds ratios (ORs) and 95% confidence intervals (95% CIs). The model evaluated the association between lifetime dietary supplement use and self-rated knowledge, information source, fitness beliefs, and favourability towards informative meetings. Adjustments were made for age group, sex, smoking status, degree course level, and field of study. Ref—Reference categories. The vertical dashed line indicates the null value (OR = 1); horizontal segments represent the 95% confidence intervals and the markers the point estimates, plotted on a logarithmic scale.

Furthermore, higher odds of use were reported among participants consulting specific sources of information, compared to those with no information, particularly health professionals (OR = 2.2; 95% CI: 1.6–3.0), friends or acquaintances (OR = 1.9; 95% CI: 1.4–2.6), and the internet or social media (OR = 1.6; 95% CI: 1.2–2.1). A significant association was observed for enrolment in scientific or technological fields (OR = 1.7; 95% CI: 1.2–2.3) (Figure 1).

**Figure 2:**
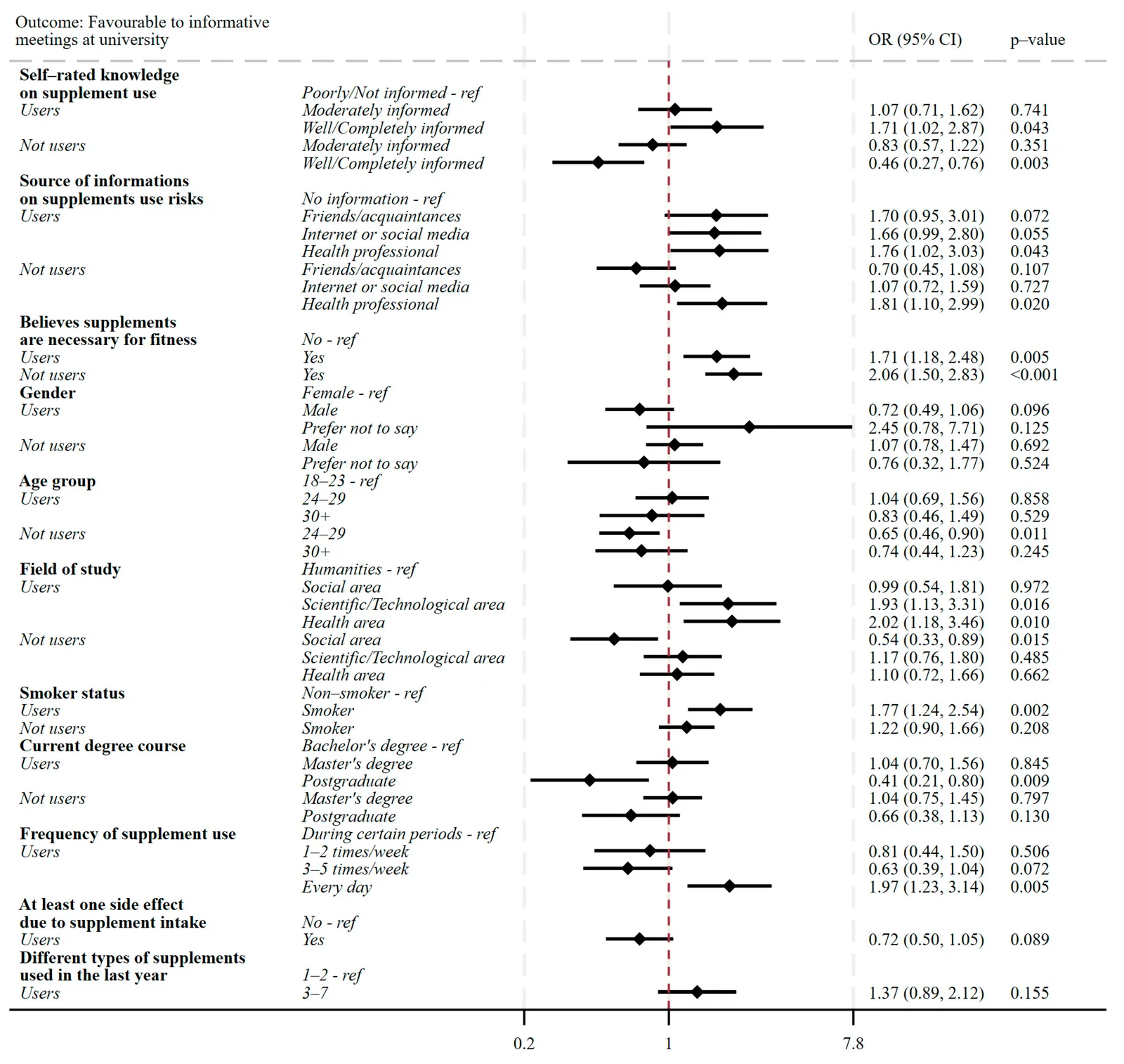
Multivariable logistic regression analysis of factors associated with favourability towards university-based informative meetings, stratified by lifetime dietary supplement use (full sample of respondents). Data are presented as adjusted odds ratios (ORs) and 95% confidence intervals (95% CIs). Separate models were fitted for dietary supplement users and non-users. Ref—Reference categories. The vertical dashed line indicates the null value (OR = 1); horizontal segments represent the 95% confidence intervals and the markers the point estimates, plotted on a logarithmic scale.

Subgroup analyses were conducted to investigate factors associated with favourability towards university-based informative meetings. The sample was stratified by lifetime DS use. Among DS users, favourability towards informative meetings was positively associated with being well or completely informed (OR = 1.7; 95% CI: 1.0–2.9) and receiving information from healthcare professionals (OR = 1.8; 95% CI: 1.0–3.0). Higher odds were observed among participants who believe supplements are necessary for fitness (OR = 1.7; 95% CI: 1.2–2.5) and those reporting daily supplement use (OR = 2.0; 95% CI: 1.2–3.1). Significant positive associations were also found for smokers (OR = 1.8; 95% CI: 1.2–2.5) and students enrolled in scientific and technological (OR = 1.9; 95% CI: 1.1–3.3) or health-related fields (OR = 2.0; 95% CI: 1.2–3.5). Conversely, a negative association was observed for postgraduate students (OR = 0.4; 95% CI: 0.2–0.8). Among non-users, favourability was positively associated with receiving information from health professionals (OR = 1.8; 95% CI: 1.1–3.0) and the belief that supplements are necessary for fitness (OR = 2.1; 95% CI: 1.5–2.8). Conversely, negative associations were observed for participants self-rating their knowledge as well-informed or completely informed (OR = 0.5; 95% CI: 0.3–0.8). Lower odds were also found for students enrolled in social fields (OR = 0.5; 95% CI: 0.3–0.9) and individuals aged 24–29 years (OR = 0.7; 95% CI: 0.5–0.9) (Figure 2).

## 4. Discussion

The current cross-sectional study assessed the lifetime prevalence and the factors associated with the use of DSs among university students in Catania. The observed lifetime prevalence of DS use, which was 55.7%, is consistent with the broader, systemic escalation in DS consumption reported in the existing literature among young adults [9,10,11,12]. Most users reported one to two supplement types, and research consistently indicates a progressive cultural institutionalization of self-supplementation [13]. In previous literature, use has been related to perceived health maintenance, cognitive/academic performance, and physical appearance goals, as well as to peer influence, personal beliefs, and social media exposure [10,12,25,26,27].

A marked gender disparity emerged, with higher use among males, consistent with the literature describing males as more inclined toward performance-oriented products (protein, amino acids, creatine), whereas females more typically use multivitamins or “beauty” supplements [13,28].

Participants who perceived themselves as moderately or highly informed were up to four times more likely to consume DSs. As objective knowledge was not assessed, this finding concerns perceived rather than actual competence. Perceived risk–benefit judgements may be central to this pattern: students who consider themselves well-informed tend to regard supplements as advantageous, safe, or even indispensable, thereby motivating their use [11]. This may suggest, although untested, a bias akin to the Dunning–Kruger effect, whereby limited exposure to nutritional science fosters overconfidence in managing complex supplementation regimens [29]. Consistently, internet and social media emerged as the predominant sources of information on supplement-related risks, alongside personal trainers; consultation with health professionals, although associated with higher odds of use, was less common, reflecting the largely unregulated nature of online nutritional content [30]. High daily exposure to idealized bodies and supplement-heavy lifestyles on social media likely reinforces perceived norms and pressure around body image [31,32]. Advice provided within the gym environment may also be perceived as accessible and aligned with appearance or performance goals. Although personal trainers have a recognized role in exercise programming, recommending multi-ingredient DSs falls outside their scope of practice, which may increase the risk of adverse drug–nutrient interactions [33,34].

Regarding academic disciplines, students in scientific and technological fields were more likely to report DS use, whereas enrolment in health-related fields was not independently associated with use. Research nonetheless suggests that health-related curricula enhance students’ DS-related knowledge, particularly among pharmacy students, and encourage reliance on evidence-based sources such as professional literature and health experts [11,35,36].

Another notable finding concerns cigarette smoking: smokers showed a higher crude prevalence of DS use, and among users, smoking was independently associated with greater favourability toward informative meetings. This contrasts with the “healthy user effect” described in some studies, whereby DS use tends to cluster with other health-protective behaviours, although evidence on this association remains mixed [11,37,38]. Consistent with our findings, a Midwestern U.S. university study reported that 72% of smokers were also using DSs. The researchers propose this link is indirect: smokers may use DSs as a psychological strategy to “offset” smoking and cope with cognitive dissonance, driven by the mistaken belief that supplements can neutralize tobacco’s health risks [38,39].

A further finding concerns the frequency of self-reported side effects, described by 34.6% of DS users. This proportion appears higher than that reported in other populations [10,40]. This may relate to the specific nature of supplements favoured by young adults, several of which carry a higher risk of adverse events than standard vitamin products [10,41]. Given that a fifth of users reported concurrent use of multiple supplement types, cumulative exposure may contribute to this figure, although the absence of product-level and biochemical characterization precludes any mechanistic conclusion. Interpretation nevertheless requires caution, as symptoms were self-reported, were not clinically verified and could not be attributed to a specific product. Receptivity toward university-based informative meetings was high overall (74.3%) [42], but the factors associated with it differed between users and non-users. Among users, favourability was associated with more intensive consumption behaviours (daily use and smoking status), suggesting that meetings may be perceived as most relevant by those already engaged in frequent or risk-associated use. Among non-users, favourability was instead associated with the belief that supplements are necessary for fitness, which may indicate that their interest stems from a perceived knowledge gap rather than an ongoing behaviour to address. Taken together with the frequency of self-reported side effects among users, this divergence suggests that a single, generic informational campaign may be suboptimal: content on correct use and side-effect recognition would be more relevant for current users, while content on the actual necessity of supplementation would better address non-users.

An objective appraisal of the present study requires an acknowledgment of both its methodological strengths and inherent limitations. The primary strength of the investigation is the robust sample size of 2001 participants, a large sample that provides adequate statistical power to detect demographic and behavioural variations across diverse academic strata and multivariable analyses allowing for nuanced identification of covariates and understanding of the precise covariates driving both supplement consumption and educational receptivity. However, the cross-sectional design precludes the establishment of definitive causal relationships between the observed variables; for instance, it cannot be established whether the belief that supplements are necessary for fitness precedes consumption or serves as a post hoc rationalization, nor whether supplement use precedes or follows interest in educational initiatives. Participants were recruited through non-probability convenience sampling via an open link, so the sampling frame and the response rate could not be determined, and the sample may over-represent students interested in nutrition or physical activity; the findings should therefore be read as descriptive of this large student sample rather than as population-level estimates. Self-rated knowledge was assessed by self-reporting; no objective knowledge test was administered, and the supplement section of the questionnaire was purpose-built and not formally validated. The reliance on a self-administered, web-based questionnaire also introduces potential recall and social desirability bias, and as a single-centre study conducted at one university, the findings may not be generalizable to the wider Italian student population. Finally, DSs were aggregated into broad self-reported categories without biochemical classification, and reported symptoms were neither clinically verified nor linked to specific products, so the study was not designed to quantify the safety profile of individual products. Despite these limitations, the study supports the relevance of integrating formal, evidence-based nutritional education into the university curriculum as a public health measure.

## 5. Conclusions

In summary, this investigation describes a high lifetime prevalence of DS use, associated primarily with self-rated knowledge, digital information channels, and fitness-related beliefs. This study analyzed a large, single-centre Italian university population, describing a discrepancy between high self-rated knowledge and a 34.6% rate of self-reported side effects, and an association between smoking and both supplement use and educational receptivity. Notably, the factors associated with favourability toward informative meetings differed between users and non-users: users’ interest was associated with more intensive consumption behaviours, while non-users’ interest was associated with fitness-related beliefs, pointing to the need for differentiated, rather than one-size-fits-all, educational strategies. Building upon these key findings, future research should prioritize larger-scale studies to deeply investigate the specific categories of supplements being consumed and thoroughly evaluate their associated side effects. Furthermore, longitudinal designs and biochemical classifications would be required to identify the compounds associated with the reported side effects. Given the high receptivity toward institutional initiatives, these findings underscore the need for universities to integrate formal, evidence-based nutritional education into their curricula, differentiated for users and non-users according to their distinct informational needs and concerns, to mitigate the risks of unregulated advice and safeguard the physiological well-being of the student population.

## Figures and Tables

**Table 1:** Sociodemographic, academic, and opinion-related characteristics of the study population (survey respondents, N = 2001), stratified by lifetime dietary supplement use. Data are presented as absolute frequencies (N) and column percentages (%). Differences between users and non-users of DSs were evaluated using Pearson’s chi-squared test.

Variable	N.		Dietary Supplement Users (%)		Dietary Supplement Non-Users (%)		*p*-Value
**Ever use dietary supplements**	2001		1114	(55.7)	887	(44.3)	
**Age group**							0.291
18–23	1059	(52.9)	587	(52.7)	472	(53.2)	
24–29	759	(37.9)	434	(39.0)	325	(36.6)	
30+	183	(9.2)	93	(8.3)	90	(10.1)	
**Gender**							<0.001
Female	1016	(50.8)	507	(45.5)	509	(57.4)	
Male	927	(46.3)	576	(51.7)	351	(39.6)	
Prefer not to say	58	(2.9)	31	(2.8)	27	(3.0)	
**Smoker status**							<0.001
Non-smoker	974	(48.7)	502	(45.1)	472	(53.2)	
Smoker	1027	(51.3)	612	(54.9)	415	(46.8)	
**Current degree course**							0.054
Bachelor’s degree	1071	(53.5)	614	(55.1)	457	(51.5)	
Master’s degree	787	(39.3)	433	(38.9)	354	(39.9)	
Postgraduate (Specialization, Master, PhD)	143	(7.2)	67	(6.0)	76	(8.6)	
**Field of study**							<0.001
Humanities	301	(15)	118	(10.6)	183	(20.6)	
Social area	243	(12.1)	121	(10.9)	122	(13.7)	
Scientific/Technological area	714	(35.7)	489	(43.9)	225	(25.4)	
Health area	743	(37.1)	386	(34.6)	357	(40.2)	
**Source of information on supplement use risks**							<0.001
No information	485	(24.2)	140	(12.6)	345	(38.9)	
Friends/acquaintances	424	(21.2)	283	(25.4)	141	(15.9)	
Internet or social media	679	(34.0)	419	(37.6)	260	(29.3)	
Health professional	413	(20.6)	272	(24.4)	141	(15.9)	
**Self-rated knowledge on supplement use**							<0.001
Not informed at all	371	(18.5)	99	(8.9)	272	(30.7)	
Poorly informed	463	(23.1)	165	(14.8)	298	(33.6)	
Moderately informed	840	(42.0)	611	(54.8)	229	(25.8)	
Well informed	267	(13.3)	201	(18.0)	66	(7.4)	
Completely informed	60	(3.0)	38	(3.4)	22	(2.5)	
**Believe supplements are necessary for fitness**							<0.001
No	838	(41.9)	357	(32.1)	481	(54.2)	
Yes	1163	(58.1)	757	(67.9)	406	(45.8)	
**Favourable to informative meetings at university**							<0.001
No	514	(25.7)	201	(18.0)	313	(35.3)	
Yes	1487	(74.3)	913	(82.0)	574	(64.7)	

**Table 2:** Use-related characteristics among university students reporting lifetime use of dietary supplements (subgroup of DS users). Data are presented as absolute frequencies (n) and column percentages (%).

Variable	N.	%
**Different types of supplements used in the last year**	1114	
1–2	866	77.7
3–7	248	22.3
**Frequency of supplement use**		
Only during certain periods	247	22.2
1–2 times/week	75	6.7
3–5 times/week	197	17.7
Every day	595	53.4
**Have experienced at least one side effect following supplement intake**		
No	729	65.4
Yes	385	34.6
**Who recommended supplement use**		
Self-taught	215	19.3
Friend/training partner	207	18.6
Doctor	95	8.5
Nutritionist/dietitian	244	21.9
Personal trainer	353	31.7

## Data Availability

The data presented in this study are not publicly available due to ethical and privacy restrictions. Data are available from the corresponding author upon reasonable request and with permission of the local Ethics Committee.

## Supplementary Materials

The following supporting information can be downloaded at: https://www.mdpi.com/article/10.3390/nu18172760/s1, File S1. Study questionnaire.
