# Supplementary material for: Prevalence and Correlates of Dietary Supplement Use Among Italian University Students: A Cross-Sectional Study
Source: Nutrients. 2026 Aug 24;18(17):2760. doi: 10.3390/nu18172760 (PMC13567380; doi:10.3390/nu18172760)
Supplement: Supplementary file 1 [file nutrients-18-02760-s001.zip › nutrients-4480630-supplementary.pdf]

Supplementary Material File S1. Study questionnaire

Introductory section - General information

0. Do you voluntarily agree to take part in this research survey?

☐ I agree
☐ I do not agree

0.1 Do you declare that you are of legal age ("The age of majority is attained on completion of the eighteenth year of age", Article 2(1) of the Italian Civil Code), that you are not pregnant, and that you are not affected by any serious condition that adversely affects your daily quality of life?

☐ Yes, I am of legal age, I am not pregnant and I am not affected by any serious condition
☐ No, I do not meet the required conditions

1. Age

☐ 18-23
☐ 24-29
☐ 30+

2. Sex

☐ Male
☐ Female
☐ Prefer not to answer

3. Course currently attended (undergraduate/postgraduate)

☐ Bachelor's degree
☐ Master's degree
☐ Specialisation school / second-level master's course / PhD

4. Highest educational qualification obtained

☐ Upper secondary school diploma
☐ Bachelor's degree
☐ Master's degree or higher

5. Field of study

☐ Health sciences
☐ Science and technology
☐ Social sciences
☐ Humanities

6. How often do you usually engage in physical activity during the week?

☐ 0-1 times per week
☐ 2-3 times per week
☐ 4-6 times per week
☐ More than 6 times per week

7. Type of physical activity mainly practised

☐ Bodybuilding / resistance training
☐ Cardio / fitness
☐ Group classes
☐ Mixed training

☐ Other

8. Are you a smoker?

☐ Yes
☐ No

9. Residence status

☐ Resident (living in the university city)
☐ Off-site student (living away from the family home)
☐ Commuter
☐ Erasmus student

10. Whom do you live with?

☐ Alone
☐ With friends or flatmates
☐ With my parents
☐ With my partner

11. Number of household members

☐ 1-3
☐ 4+

12. Use of food delivery applications (Uber Eats, Deliveroo, Just Eat, Glovo, etc.)

☐ No
☐ Rarely
☐ At least once a week

13. Use of food waste prevention applications (Too Good To Go, etc.)

☐ No
☐ Rarely
☐ At least once a week

EAT-26 - Eating Attitudes Test

Adapted from: Garner DM, Olmsted MP, Bohr Y, Garfinkel PE. The Eating Attitudes Test: psychometric features and clinical correlates. Psychol Med. 1982;12(4):871-8. doi:10.1017/s0033291700049163

Response options for all items: Always / Very often / Often / Sometimes / Rarely / Never

1. I am terrified of gaining weight.
2. When I am hungry, I avoid eating.
3. I think about food with concern.
4. I have had episodes of eating with great voracity, feeling unable to stop.
5. I have the habit of cutting my food into small pieces.
6. I pay close attention to the calorie content of the foods I eat.
7. I particularly avoid foods with a high carbohydrate content (bread, pasta, sweets).
8. I feel that others would like me to eat more.
9. I vomit after I have eaten.
10. I feel extremely guilty after eating.
11. I am tormented by the desire to be thinner.
12. I engage in intense physical exercise in order to burn calories.
13. Other people think that I am too thin.

14. I am preoccupied with the idea of having fat on my body.
15. I take longer than others to eat my meals.
16. I avoid sweet foods.
17. I eat diet foods.
18. I feel that food dominates my life.
19. I like to show great self-control around food and to master my hunger.
20. I feel that others put pressure on me to eat.
21. I devote too much time and too many thoughts to food.
22. I feel distressed if I eat sweets.
23. I follow dieting programmes.
24. I like my stomach to be empty.
25. I have the impulse to vomit after eating.
26. I enjoy trying new, elaborate foods.

Behavioural questions

Over the past six months, have you engaged in any of the following behaviours?

1. Have you had episodes of uncontrolled binge eating, in which you ate large amounts of food and felt unable to stop?

- ☐ Never
- ☐ Once a month or less
- ☐ 2-3 times a month
- ☐ Once a week
- ☐ 2-6 times a week
- ☐ Once a day or more

2. Have you made yourself vomit in order to control your weight or body shape?

- ☐ Never
- ☐ Once a month or less
- ☐ 2-3 times a month
- ☐ Once a week
- ☐ 2-6 times a week
- ☐ Once a day or more

3. Have you used laxatives, diuretics or diet pills in order to control your weight or body shape?

- ☐ Never
- ☐ Once a month or less
- ☐ 2-3 times a month
- ☐ Once a week
- ☐ 2-6 times a week
- ☐ Once a day or more

4. Over the past six months, have you trained for more than 60 minutes a day in order to lose weight or control your body shape?

- ☐ Never
- ☐ Once a month or less
- ☐ 2-3 times a month
- ☐ Once a week
- ☐ 2-6 times a week

☐ Once a day or more

5. Over the past six months, have you lost a large amount of weight (10 kg or more)?

☐ Yes
☐ No

Section SA - Dietary supplement use

6. Have you ever used dietary supplements?

☐ Yes
☐ No (if so, go directly to question 14)

7. If yes, which types of supplements have you used over the past year? (more than one answer may be selected)

☐ Protein powders (e.g. whey)
☐ Creatine
☐ Essential amino acids / BCAAs
☐ Pre-workout products
☐ Weight gainers
☐ Vitamin/mineral supplements
☐ Thermogenics

8. If yes, how often do you take these supplements?

☐ Only during certain periods
☐ 1-2 times a week
☐ 3-5 times a week
☐ Every day

9. If yes, for what reason do you use these supplements?

☐ To increase muscle mass
☐ Definition / cutting
☐ Post-exercise recovery
☐ To enhance performance
☐ As a meal replacement
☐ Recommended by friends / a personal trainer

10. If yes, who advised you to use them?

☐ No one, self-directed
☐ A friend or training partner
☐ A personal trainer
☐ A nutritionist or dietitian
☐ A physician

11. If yes, have you ever experienced adverse effects after taking supplements? (more than one answer may be selected)

☐ Gastrointestinal disturbances
☐ Headache
☐ Insomnia
☐ Palpitations or tachycardia
☐ Kidney or liver problems

☐ Mood alterations
☐ No adverse effects

Section SB - Awareness and health

12. Have you ever received information about the risks associated with the improper use of dietary supplements?

☐ Yes, from a healthcare professional
☐ Yes, from the internet or social media
☐ Yes, from friends or acquaintances
☐ No

13. How well informed do you consider yourself to be about the correct use of dietary supplements?

☐ Not at all informed
☐ Slightly informed
☐ Moderately informed
☐ Well informed
☐ Fully informed

Section SC - Opinions and prevention

14. Do you believe that the use of dietary supplements is necessary in order to improve physical fitness?

☐ Yes
☐ No

15. Would you be in favour of information sessions or free counselling on dietary supplements at the sports centre?

☐ Yes
☐ No

MDDI - Muscle Dysmorphic Disorder Inventory

Adapted from: Santarnecchi E, Dèttore D. Muscle dysmorphia in different degrees of bodybuilding activities: validation of the Italian version of Muscle Dysmorphia Disorder Inventory and Bodybuilder Image Grid. Body Image. 2012;9(3):396-403. doi:10.1016/j.bodyim.2012.03.006

Response options for all items: Never / Rarely / Sometimes / Often / Always

1. I think my body is too slight.
2. I wear loose clothing so that people cannot see my body.
3. I hate my body.
4. I would like to become bigger and more massive.
5. I think my chest is too small.
6. I think my legs are too thin.
7. I feel as though I have too much fat on my body.
8. I wish my arms were bigger.
9. I feel ashamed to be seen without a shirt / T-shirt / etc.
10. I feel anxious (uneasy) when I miss one or more training sessions.
11. I neglect social activities with friends in order to keep to my training programme.
12. I feel low in mood when I miss one or more training sessions.
13. I miss opportunities to make new friends in order to keep to my training programme.

MEDAS - Mediterranean Diet Adherence Screener

Adapted from: Estruch R, Ros E, Salas-Salvadó J, et al. Primary prevention of cardiovascular disease with a Mediterranean diet supplemented with extra-virgin olive oil or nuts. N Engl J Med. 2018;378(25):e34. doi:10.1056/NEJMoa1800389

1. Do you use olive oil as your main culinary fat?

☐ Yes
☐ No

2. How much olive oil do you consume in a day (including that used for frying, dressing salads, meals eaten away from home, etc.)?

☐ 4 or more tablespoons
☐ Fewer than 4 tablespoons

3. How many servings of vegetables do you consume per day? (1 serving = 200 g; count side dishes as ½ serving)

☐ 2 or more servings (at least 1 of them raw or as salad)
☐ Fewer than 2 servings

4. How many units of fruit (including natural fruit juices) do you consume per day?

☐ 3 or more units
☐ Fewer than 3 units

5. How many servings of red meat, hamburgers or meat products (ham, sausage, etc.) do you consume per day? (1 serving = 100-150 g)

☐ Fewer than 1 serving
☐ 1 or more servings

6. How many servings of butter, margarine or cream do you consume per day? (1 serving = 12 g)

☐ Fewer than 1 serving
☐ 1 or more servings

7. How many sweetened or carbonated beverages do you drink per day?

☐ Fewer than 1 glass
☐ 1 or more glasses

8. How much wine do you drink per week?

☐ 7 or more glasses
☐ Fewer than 7 glasses

9. How many servings of pulses do you consume per week? (1 serving = 150 g)

☐ 3 or more servings
☐ Fewer than 3 servings

10. How many servings of fish or seafood do you consume per week? (1 serving = 100-150 g of fish, or 4-5 units or 200 g of seafood)

☐ 3 or more servings
☐ Fewer than 3 servings

11. How many times per week do you consume commercial (not home-made) sweets or pastries, such as cakes, biscuits, snack cakes or puddings?

☐ Fewer than 3 times
☐ 3 or more times

12. How many servings of nuts (including peanuts) do you consume per week? (1 serving = 30 g)

- ☐ 3 or more servings
- ☐ Fewer than 3 servings

13. Do you prefer to eat chicken, turkey or rabbit instead of veal, pork, hamburgers or sausage?

- ☐ Yes
- ☐ No
- ☐ I do not eat meat

14. How many times per week do you consume vegetables, pasta, rice or other dishes seasoned with sofrito (a sauce made with tomato and onion, leek or garlic, simmered in olive oil)?

- ☐ 2 or more times
- ☐ Fewer than 2 times
